# A Case Report of Hemophagocytic Lymphohistiocytosis and Meningitis Due to Atezolizumab Treatment for Lung Adenocarcinoma

**DOI:** 10.7759/cureus.58253

**Published:** 2024-04-14

**Authors:** Hiroaki Ota, Miyuki Munechika, Kazunori Tobino, Kazuki Uchida, Yosuke Muarakami

**Affiliations:** 1 Respiratory Medicine, Iizuka Hospital, Iizuka, JPN

**Keywords:** ici, meningitis, hlh, irae, atezolizumab

## Abstract

Immune checkpoint inhibitors (ICIs) are used to treat a variety of tumors. Despite their broad beneficial effects, these inhibitors can cause immune-related adverse events (irAEs) and even death. Hemophagocytic lymphohistiocytosis (HLH) and meningitis, although infrequent, can be aggressive and life-threatening due to excessive immune activation. Herein, we report a case of an 80-year-old man who developed HLH after receiving atezolizumab monotherapy as a second-line treatment for lung adenocarcinoma. He was treated for HLH with oral prednisolone (PSL), but further ataxia and dysuria developed, and a lumbar puncture diagnosed meningitis. Both HLH and meningitis improved with continued oral PSL treatment. This is the first case of atezolizumab-induced HLH with meningitis and highlights the importance of early diagnosis and treatment for rare irAE.

## Introduction

Immune checkpoint inhibitors (ICIs) have revolutionized the treatment of various cancers, including lung adenocarcinoma. However, these agents can lead to immune-related adverse events (irAEs) due to their mechanism of action, which involves enhancing the immune system's response against tumor cells [1]. While irAEs can affect any organ system, some of the most severe and potentially life-threatening events include hemophagocytic lymphohistiocytosis (HLH) and central nervous system (CNS)-related adverse events (AEs), such as meningitis [2]. HLH is a rare but fatal hyperinflammatory syndrome characterized by excessive activation of macrophages and T lymphocytes, leading to cytokine storms and multiorgan failure [3]. The incidence of HLH as an irAE is extremely low, with only a few case reports and series documented in the literature [4,5]. A systematic review by Dupré et al. found only 20 cases of HLH associated with ICI [6]. Similarly, CNS-related AEs, including meningitis, are uncommon but serious complications of ICI therapy. The most common neurological AEs were encephalopathy, meningitis, Guillain-Barré-like syndrome, and myasthenia syndrome. A meta-analysis by Cuzzubbo et al. found that the overall incidence of neurological AEs was 3.8% with anti-CTLA4 antibodies, 6.1% with anti-PD1 antibodies, and 12.0% with the combination of both [7]. The incidence of high-grade neurological AEs was less than 1% for all ICI treatments. The co-occurrence of HLH and meningitis as irAEs is an exceptionally rare phenomenon. To date, only one case report has described a patient who developed both HLH and meningitis during combination therapy with ipilimumab and nivolumab for malignant melanoma [8]. The patient's meningitis improved with systemic corticosteroid treatment, highlighting the importance of prompt recognition and management of these complications. To the best of our knowledge, there have been no reported cases of concurrent HLH and meningitis associated with atezolizumab monotherapy in the treatment of lung adenocarcinoma. This scarcity of literature underscores the need for heightened awareness and vigilance among clinicians when using ICIs, as early detection and intervention can significantly improve patient outcomes [9]. In this report, we present a unique case of a patient with lung adenocarcinoma who developed both HLH and meningitis during treatment with atezolizumab. The patient was successfully managed with systemic corticosteroid therapy, emphasizing the critical role of timely diagnosis and appropriate treatment in the management of these rare but potentially life-threatening irAEs.

## Case presentation

An 80-year-old Japanese man was referred to our hospital in November 2018 with complaints of food intake difficulty and myalgia. He had been diagnosed with lung adenocarcinoma (Stage IIIA: T4N0M0, EGFR gene mutation-negative, EML-ALK fusion gene-negative, and PD-L1 TPS <1%) one year earlier. He did not have sufficient lung function to undergo surgery or radiation therapy because of chronic obstructive pulmonary disease (COPD) and was started on chemotherapy with carboplatin (CBDCA), pemetrexed (PEM), and bevacizumab (Bev). After four cycles of this chemotherapy with a significant response, followed by 11 cycles of maintenance therapy with PEM and Bev, the lung cancer grew again. Therefore, atezolizumab (an immune checkpoint inhibitor, ICI) monotherapy was started as second-line treatment. Ten days after atezolizumab administration, fever (39°C), myalgia, and anorexia occurred. Upon experiencing symptoms for a period of five days, he was admitted to the hospital for further examination and treatment.

He had a history of glaucoma, gastric ulcers, and COPD. He smoked 45 pack-years between the ages of 25 and 70 and drank one drink per day. He had no allergies. His initial vital signs were as follows: oxygen saturation on room air, 98%; body temperature, 36.5 ℃; blood pressure, 158/58 mmHg; pulse rate, 82/min; respiratory rate, 20/min; and level of consciousness, clear. On physical examination, the heart sounds were normal, and the lungs were clear. No other specific findings were noted. Laboratory tests showed decreased white blood cell count (2,680/μL) and platelet count (35,000/μL), and increased LDH (672 U/L) and ferritin (3,318 ng/mL) levels (Table 1).

**Table 1:** Laboratory findings on admission ALT: Alanine aminotransferase, BUN: Blood urea nitrogen, CRP: C-reactive protein, sIL2R: Soluble interleukin-2 receptor

Blood analyses	Result	Reference range
Hemoglobin (g/dL)	15.7	13.7-16.8
White blood cells (/μL)	2680	3,300-8,600
Neutrophil (%)	65	40-75
Lymphocyte (%)	23	21-54
Platelets (/μL)	35000	158,000-348,000
AST (U/L)	39	13-30
ALT (U/L)	32	10-42
BUN (mg/dL)	10	8-20
Creatinine (mg/dL)	0.43	0.65-1.07
LDH (U/L)	672	124-222
CRP (mg/dL)	5.24	<0.14
Ferritin (ng/mL)	3318	10-250
Triglycerides (mg/dL)	129	40-234
Fibrinogen (mg/dL)	510.6	168-327
D-dimer (μg/mL)	37.4	≦1.0
sIL2R (U/mL)	1432	121-613

**Figure 1:**
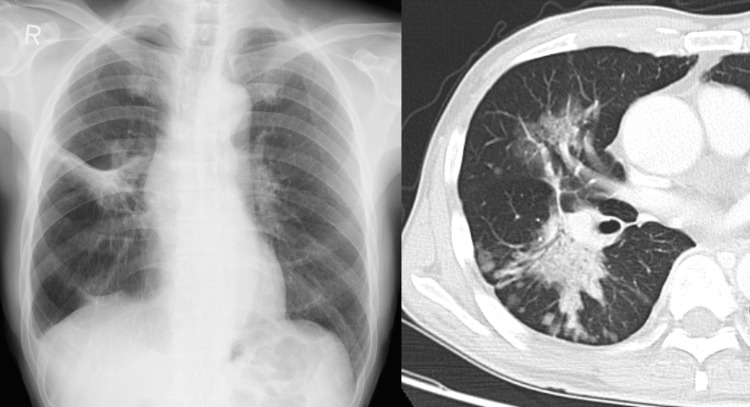
Chest radiography and CT images on admission Ground-glass opacity on the right middle lung lobe and consolidation on the right lower lung lobe with multiple patchy ground-glass opacities in its periphery were observed.

Chest radiography and computed tomography (CT) revealed ground-glass opacity on the right middle lung lobe and consolidation on the right lower lung lobe with multiple patchy ground-glass opacities in its periphery (Figure 1).

**Figure 2:**
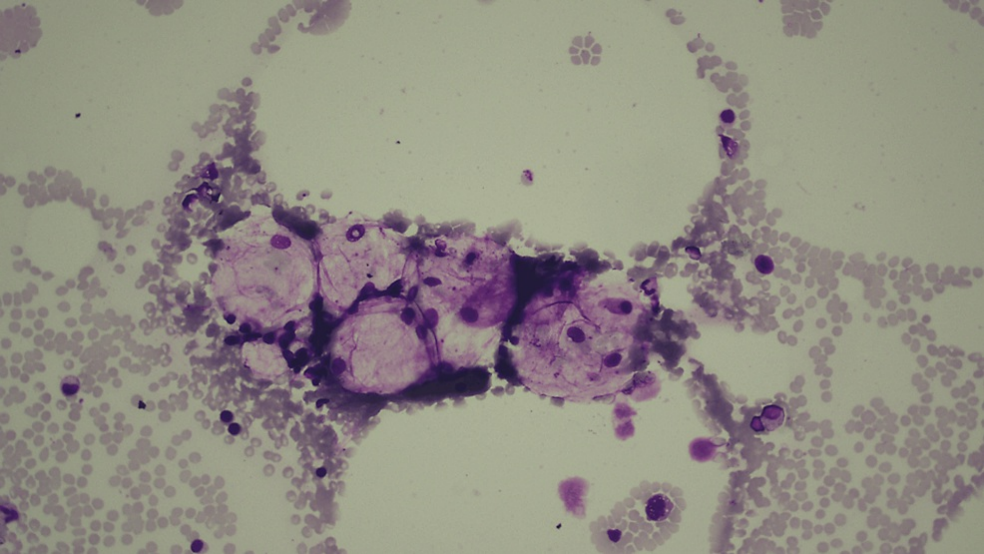
Image of bone marrow aspiration Histiocyte hypoplasia and phagocytosis of erythrocytes, leukocytes, and platelets by macrophages were noted.

These lesions were previously recognized lung cancers and did not change. Sputum stains and cultures, urinary antigen tests, and blood cultures did not detect any obvious pneumonia-causing organisms. Bone marrow aspiration revealed histiocyte hypoplasia and increased macrophage engulfing of erythrocytes, leukocytes, and platelets (Figure 2).

**Figure 3:**
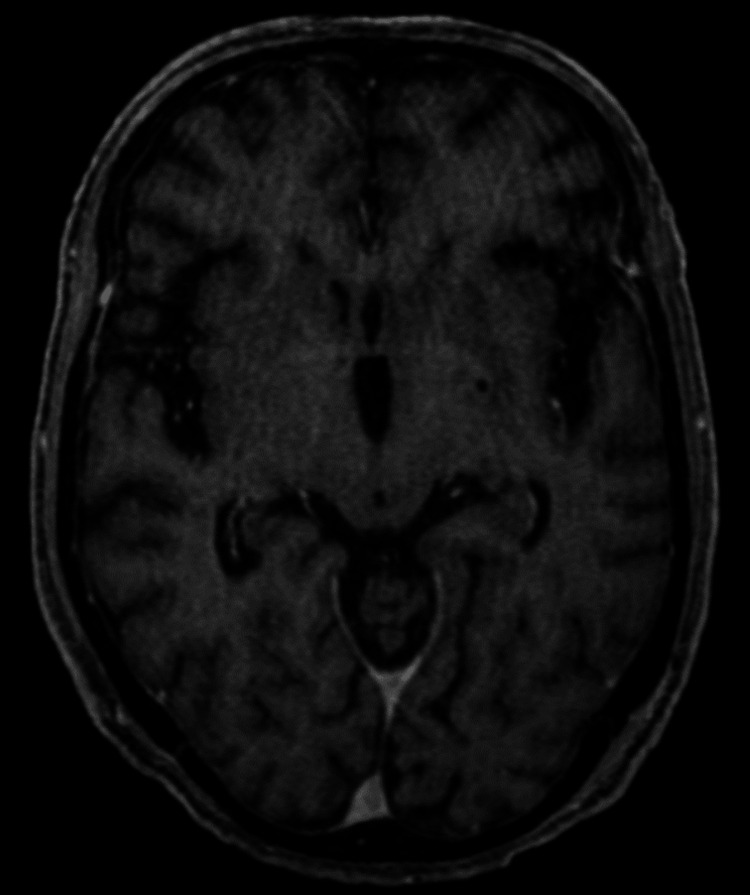
Contrast brain MRI T1-weighted image No findings indicative of encephalitis or other brain disease were noted.

Based on these results and the timing of the atezolizumab administration, we determined that the patient had HLH as irAE on day three of admission. Prednisolone (PSL) 50 mg/day (1 mg/kg/day) was started on the day of admission, and soon thereafter blood counts recovered, LDH decreased, and symptoms improved. Ataxia and dysuria occurred on day seven, but there were no specific head CT and MRI findings (Figure 3).

A lumbar puncture was performed, and the results were as follows: initial pressure of 6 cmH2O, cell count of 49/3 mm^3^ (0% polynuclear leukocytes, 100% monocytes), protein of 126 mg/dL, sugar of 67 mg/dL, and Cl of 116 mEq/L. No bacteria were found in the spinal fluid, and the ink stain was negative, and spinal fluid PCR for meningitis-causing virus and tuberculosis was also negative. Although there were no physical findings such as headache or rigidity of the neck, the spinal fluid findings were suggestive of aseptic meningitis; meningitis as irAE is often difficult to differentiate from encephalitis, and since there were no findings suggestive of encephalitis on MRI, we diagnosed this patient with meningitis. The dose of PSL was started at 50 mg/day and was reduced to 40 mg/day of PSL on the seventh day because the fever resolved after six days of admission and blood cells showed a recovery trend. On the same day, the patient developed meningitis, but his symptoms gradually improved from day 8 to 11, so the PSL dose was maintained, and the dose was reduced to 30 mg/day on day 12. The PSL dose was then reduced by 5 mg/day every two weeks and discontinued five months after the start of steroid therapy. During that time, symptoms of irAE did not recur. In parallel, he was treated with cytotoxic anticancer drugs, but his lung cancer continued to worsen, and he died two years after the onset of HLH.

## Discussion

Here, we presented a case of hemophagocytic lymphohistiocytosis and meningitis due to atezolizumab treatment for lung adenocarcinoma. Notably, both conditions exhibited significant improvement with systemic corticosteroid therapy. It is noteworthy to mention that, as of February 2024, VigiBase (http://www.vigiaccess.org/) has only registered 18 cases of HLH and two cases of meningitis resulting from the anti-PD-L1 antibody atezolizumab, and there have been no reports of concomitant HLH and meningitis attributable to atezolizumab.

In the IMpower150 study, the incidence of meningitis was 0.25% (one of 393 patients) and no HLH [10]. In the OAK study, the incidence of encephalitis and meningitis was 0.8% (five of 609 patients) in the atezolizumab group, while in the Japanese population, this rate was 7.1% (four of 56 patients), indicating a higher incidence in Japanese patients [11]. To the best of our knowledge, at this time, no studies on racial differences in encephalitis or meningitis as irAE have been reported and are not clear. Perhaps the widespread use of CT and MRI in Japan makes the diagnosis of these diseases easier than in other countries. Further case accumulation and comparative studies are needed. In the same study, none of the patients in the atezolizumab group developed HLH. Therefore, the combination of HLH and meningitis due to atezolizumab is very rare, making this case very valuable. To the best of our knowledge, there have been two previous case reports of HLH due to atezolizumab, with two cases reported [12,13]. Due to the small number of reports of atezolizumab-induced HLH, the predisposing factors for this disease have not yet been clearly identified. However, the reported rates of ICI (including atezolizumab)-associated HLH are particularly high in France, Germany, and Japan, suggesting that genetic factors may be involved [14]. Furthermore, HLH has been reported more frequently in melanoma cases in which the use of ipilimumab plus nivolumab was approved earlier, suggesting that the combination of CTLA-4 antibodies may increase the incidence. Noseda et al. reported that HLH occurs 6.7 weeks (IQR 2.9-15.4, n=18) after the start of treatment with ICI [14]. In addition to their report, we reviewed 10 other case reports (11 patients) that have been reported so far; four of 11 patients had exacerbations of collagen disease or other irAEs; one patient had somnolence and inability to speak, but only nonspecific findings on EEG; no meningitis occurred in any patient. All patients were treated with steroids, including six with methylprednisolone (mPSL) pulse, four with etoposide, and one with tocilizumab. One patient died; others were discharged with mild symptoms (Table 2) [12,13,15-21].

**Table 2:** Case reports of HLH caused by immune checkpoint inhibitors CBDCA, carboplatin; mPSL, methylprednisolone; N.A., not applicable; PSL, prednisolone

Reports	Age/Sex	Histology	PD-L1	Treatment (cycles) before HLH occurs	IrAE other than HLH	Treatment for HLH	Central nervous system symptoms	Clinical course
Okawa et al. [15]	78/Maleale	Squamous cell lung cancer	N.A.	Pembrolizumab (1 cycle)	Autoimmune hemolytic anemia	mPSL pulse	None	Improved
Akagi et al. [16]	74/Male	Lung adenocarcinoma	60%	Pembrolizumab (1 cycle)	Worsening of rheumatoid arthritis	mPSL pulse, dexamethasone with etoposide	None	Improved
Kurozumi et al. [17]	75/Male	Lung adenocarcinoma	N.A.	Pembrolizumab (unknown)	None	mPSL pulse	None	Improved
	60/Female	Lung adenocarcinoma	N.A.	Pemetrexed with Pembrolizumab (2 cycles)	None	mPSL pulse	None	Improved
Rubio-Perez et al. [12]	67/Male	Lung adenocarcinoma	＜1％	Atezolizumab (unknown)	None	Dexamethasone, tocilizumab, anakinra, mycophenolate mofetil, and etoposide	None	Dead
Wei et al. [18]	50/Female	Thymic cancer	N.A.	Pembrolizumab (unknown)	None	Dexamethasone with etoposide	None	Improved
	70/Male	Squamous cell lung cancer	N.A.	Pembrolizumab (unknown)	None	PSL	None	Improved
Oyama et al. [19]	60/Male	Lung adenocarcinoma	N.A.	CBDCA + Pemetrexed + Pembrolizumab (unknown) followed by Pemetrexed with Pembrolizumab (unknown)	None	PSL	None	Improved
Sackstein et al. [20]	55/Male	Lung adenocarcinoma	N.A.	CBDCA + Pemetrexed + Pembrolizumab (3 cycles)	None	mPSL pulse, mPSL with tosilizumab	Somnolence, difficulty speaking	Improved
Endo et al. [13]	65/Female	Lung adenocarcinoma	N.A.	CBDCA + Pemetrexed + Atezolizumab (unknown)	Autoimmune hemolytic anemia	PSL	None	Improved
Honjo et al. [21]	52/Male	Lung adenocarcinoma	N.A.	Nivolumab (unknown)	Cytokine release syndrome	mPSL, PSL with mycophenolate mofetil	None	Improved

Our patient showed mild improvement after the onset of meningitis, without any change in steroid dosage. In cases of meningitis, an increase in mPSL of 1000 mg may be considered if the grade is significant. This patient had already started PSL therapy for HPS and may not have been severely ill.

About meningitis caused by atezolizumab, there have been five reported cases in the past [22-24]. The number of reported cases was still small, and the characteristics of patients susceptible to meningitis were unknown. The mean time from administration to onset of meningitis is reported to be three months for all ICIs, although meningitis caused by atezolizumab has been reported to occur within three weeks after administration in patients with advanced NSCLC, with or without concurrent chemotherapy. The onset of meningitis symptoms caused by atezolizumab in our patient occurred earlier than meningitis caused by other ICIs, at 17 days after administration. However, the diagnosis of meningitis is not easy and may be difficult in some facilities, as it requires spinal fluid testing, electroencephalography, and head MRI testing based on the diagnostic criteria established by the International Encephalitis Consortium [25]. In addition, HLH itself is said to cause CNS symptoms, and reports in adults indicate that CNS symptoms appear in 10-70% of patients after the onset of HLH [26]. The neurological symptoms in HLH are thought to be caused by abnormal production of inflammatory cytokines, called cytokinin storms, that alter inflammatory responses and neuroplasticity as effects on the central nervous system. Because CNS symptoms are nonspecific, it is difficult to clinically distinguish between the two. In our patient, neurologic symptoms developed while laboratory findings related to HLH improved after PSL administration was started. In addition, since the MRI showed no findings suggestive of encephalitis and the CSF findings were strongly suggestive of aseptic meningitis, meningitis was determined to be irAE rather than neurological findings associated with HLH. Treatment of immune-associated meningitis involves discontinuing the use of the associated ICI and administering corticosteroids in the range of 0.5 mg/kg to 1,000 mg mPSL daily. In severe cases, the use of antivirals, antibacterials, intravenous immunoglobulin (IVIG), and plasmapheresis may also be considered. Many patients have reported improvement with corticosteroid therapy, and our patients improved soon after starting that therapy. There has been a case report of a patient who developed meningitis during PSL treatment for HLH caused by combination therapy with ipilimumab and nivolumab for malignant melanoma, and the patient's meningitis improved when the PSL was changed to mPSL and the dose increased [8]. Therefore, this is the first report of simultaneous HLH and meningitis caused by atezolizumab. Throughout this patients, the following points were considered important: (1) immune-related meningitis can occur in patients receiving corticosteroid therapy, but treatment depends on the severity of the disease; and (2) in severe cases, the corticosteroid dosage should be increased, but not in mild cases.

## Conclusions

In conclusion, we report the first case of concurrent HLH and meningitis as irAEs associated with atezolizumab monotherapy in a patient with lung adenocarcinoma. HLH and meningitis are both uncommon but severe irAEs that can occur with ICI therapy. The co-occurrence of these two conditions is even rarer, with only one previous case reported in the literature, which was associated with combination therapy using ipilimumab and nivolumab. The early diagnosis of HLH and meningitis can be challenging due to their nonspecific symptoms and the lack of awareness among clinicians regarding these rare irAEs. However, prompt recognition is crucial, as delayed treatment can lead to rapid clinical deterioration and potentially fatal outcomes. In our case, the timely initiation of systemic corticosteroid therapy was successful in managing both HLH and meningitis, underlining the importance of early intervention. Although corticosteroids are the mainstay of treatment for irAEs, the optimal dosage and duration of therapy for HLH and meningitis have not been well-established. In summary, this case serves as a reminder for clinicians to maintain a high index of suspicion for uncommon but serious irAEs, such as HLH and meningitis, when treating patients with atezolizumab or other ICIs. Early recognition and prompt initiation of appropriate corticosteroid therapy are paramount to improving patient outcomes and minimizing the risk of long-term sequelae.
